# Health System Stakeholder Perspectives on a Text-Based Intervention for Caregivers of Suicidal Youth 

**DOI:** 10.1007/s41347-026-00648-w

**Published:** 2026-05-23

**Authors:** Shriya Anand, Megan Chen, Valerie J. Micol, Cynthia J. Ewell Foster, Victor Y. Hong, Courtney W. Mangus, Ewa K. Czyz

**Affiliations:** Department of Psychiatry,, University of Michigan Medical School, 4250 Plymouth Rd, Ann Arbor, MI 48105 USA

**Keywords:** Text-based intervention, Suicide prevention, Caregivers, Emergency department, Implementation, Digital mental health

## Abstract

Caregivers of adolescents at elevated suicide risk are tasked with significant responsibility following their child’s Emergency Department (ED) visit, such as monitoring warning signs, restricting lethal means access, or encouraging coping. Caregiver uncertainties regarding post-discharge support, coupled with resource constraints limiting the health system’s capacity to provide ongoing assistance, call for scalable strategies to address these challenges. Digital interventions, such as text messaging, offer a pathway for low‑burden and accessible post-discharge approaches to support families. Building on a pilot of a text-based intervention for parents (TESP), developed to increase caregiver follow-through with suicide prevention recommendations and improve caregiver well-being, this qualitative mixed‑methods study sought input from health system stakeholders (clinical providers, administrative leaders and decision makers) to investigate TESP’s eventual implementation potential. Twenty-one providers and system-level staff from three EDs completed semi-structured interviews and surveys rating TESP’s acceptability, perceived feasibility, and appropriateness. A thematic framework was applied to the interview transcripts to identify salient themes. TESP was seen favorably, and ratings across the domains were moderate‑to‑high. Additional themes highlighted the intervention’s focus on validating caregivers’ experiences and reinforcing safety recommendations in a scalable way. Key implementation facilitators centered on system-level organizational structure, workflow automation, and increasing perception of value from health system staff and parents alike. The findings show TESP’s promise as a feasible, acceptable strategy to improve post-discharge safety for youth at risk for suicide, while highlighting the importance of provider and leader feedback to anticipate and address implementation barriers in resource-constrained care settings like the ED.

## Introduction

In the high-risk period following emergency department (ED) visits for suicidal thoughts or attempts, parents play a central role in maintaining their youth’s safety and well-being (Ewell Foster et al., 2025; Gay, 2025; Micol et al., 2025). During this vulnerable time, caregivers are often asked to carry out a range of suicide prevention strategies, such as monitoring suicide warning signs, securing lethal means, and facilitating follow-up care. Yet, many caregivers report feeling stressed and overwhelmed as well as uncertain about how to support their youth effectively (Czyz et al., 2018; Micol et al., 2025). At the same time, increasing pediatric ED visits for suicide risk concerns (Bommersbach et al., 2023), limited staffing, and other resource constraints make it challenging for EDs to offer ongoing support for families post discharge.

Given these challenges, scalable interventions are needed to assist caregivers and equip them with practical tools after a youth’s suicidal crisis. Digital interventions such as automated text messages offer a promising, low-burden way to extend support beyond the ED (Czyz et al., 2021; Lattie et al., 2022). To meet this need, the Text-Based Support for Parents (TESP) intervention was previously developed by several of the co-authors (see Czyz et al., 2025) to encourage caregiver engagement in recommended suicide-prevention strategies after an ED visit. Details on the intervention’s structure, content, and theoretical framework are described in the original pilot trial (Czyz et al., 2025). In brief, grounded in Self-Efficacy Theory (Bandura et al., 1977) and further informed by principles of Motivational Interviewing (Miller & Rollnick, 2012) and Self-Determination Theory (Deci & Ryan, 2004), the six-week TESP intervention was developed iteratively with input from experts and parents to provide support for parents. TESP consists of daily automated text messages targeting two interrelated components. The first component focuses on adolescent-centered (A-C) messages intended to increase parental follow-through with recommended suicide prevention activities (e.g., lethal-means restriction, monitoring warning signs) promoting a safer post-ED environment for the adolescent. The second component includes parent-centered (P-C) text messages intended to strengthen caregivers’ own well-being (e.g., coping tips) to try to increase their capacity to support their adolescent after discharge. In the pilot trial, TESP was found to be feasible and acceptable to parents who received the intervention, with post-intervention feedback suggesting parents’ overall satisfaction with the intervention and majority (over 94%) would recommend it to other caregivers. Pilot results also suggested the TESP’s preliminary impact on post-ED parental engagement in recommended prevention activities and fewer youth suicide attempts (Czyz et al., 2025). Despite these encouraging pilot findings, questions remain regarding the intervention’s broader effectiveness as well as its potential for real-world adoption.

Implementing evidence-based interventions in hospital settings remains challenging due to complex system- and staff-level barriers (Geerligs et al., 2018). Engaging health system decision makers and clinical staff to learn about potential barriers and facilitators to intervention implementation is necessary before successful adoption into care (Damschroder et al., 2009). Digital health implementation science studies similarly emphasize that early engagement helps identify workflow challenges, tailor training, and ensure scalability (Silfee et al., 2021; Lynch et al., 2025). The present study gathered perspectives from ED providers as well as administrators and leaders across two health systems on the parent-facing TESP intervention to better understand their perspectives on its acceptability, utility, and implementation potential. In addition to informing possible future integration of TESP into routine ED care, the study also offers broader insights on health system stakeholder perspectives on integrating digital suicide prevention-focused interventions into existing ED workflows.

## Methods

### Participants

Participants included ED providers as well staff in administrative or leadership roles. They were recruited via email and internal referral from three EDs across two health systems in the midwestern United States. Of the 43 individuals invited to participate in semi-structured interviews, 22 (51%) responded and 21 (49%) completed the interview. Participants included social workers (*n* = 9, 43%), physicians (*n* = 6, 29%), nurses (*n* = 4, 19%), and other staff (*n* = 2, 10%). Of the 21 participants, most identified as White (*n* = 15, 72%), followed by Asian (*n* = 4, 19%); two participants (10%) did not report race. More than half identified as female (*n* = 12, 57%), seven (33%) as male, and two (10%) did not report gender. Regarding ethnicity, 15 (71%) were non‑Hispanic/Latino, two (10%) identified as Hispanic/Latino, and four (19%) did not report their ethnicity. With respect to patient care, nearly half provided direct care between 4 and 5 days per week (*n* = 10, 48%), 33% (*n* = 7) on three days, and 10% (*n* = 2) on two or fewer days; two participants (10%) did not report this information. The study was reviewed by the Institutional Review Board and determined to be exempt. Study participation was voluntary; participants provided verbal consent to be in the study and were compensated $50 for their time.

### Semi-Structured Interview and Assessments

Participating ED providers and administrators/leaders took part in semi-structured interviews, lasting approximately 45 min, delivered with aid of interview guides developed for this study to standardize interview questions, conducted via a secure video call. Interviews were conducted by three co-authors (SA, MC, VJ), who have prior experience with carrying out semi-structured interviews and who were trained in administering the study-specific interview guide, including via mock interview practice. Interviews were transcribed using automatic transcription and were subsequently reviewed to correct any transcription errors. After providing study consent, study participants were introduced to the purpose and structure of the text-based intervention, including sample content and delivery specifications (frequency, automation, etc.). The interviews gathered qualitative feedback on the intervention components, their potential utility, as well as implementation barriers and facilitators in an ED setting. Participants also completed Likert-scale ratings assessing their perception of the intervention components and utility (see Table 1). Additional Likert-scale items, adopted from prior work addressing indicators of implementation (Proctor et al., 2011; Weiner et al., 2017) listed in Table 1, were used to assess intervention acceptability, appropriateness for caregivers of youth at risk for suicide, and perceived feasibility to implement the intervention as a follow-up to ED visits.

**Table 1:** Descriptive statistics of stakeholder perception of the intervention and its implementation potential

Question	Mean	SD	Min	Max
Perception of Intervention Components (range from 1 [dislike a lot] to 5 [like a lot])				
1. How much do you like or dislike the A-C intervention component?	4.62	0.50	4	5
2. How much do you like or dislike the P-C intervention component?	4.48	0.87	2	5
Perception of Utility (range from 1 [not at all] to 10 [very])				
3. If implemented as part of routine care in the future, how useful would the TESP intervention be as a clinical program for EDs serving these patients/families?	8.14	1.46	5	10
Perception of Acceptability (range from 1 [completely disagree] to 5 [completely agree])				
4. The TESP intervention is appealing to me.	4.65	0.49	4	5
5. I like the TESP intervention.	4.45	0.51	4	5
Perception of Appropriateness (range from 1 [completely disagree] to 5 [completely agree])				
6. The TESP intervention seems applicable.	4.45	0.60	3	5
7. The TESP intervention seems like a good match.	4.35	0.59	3	5
Perception of Feasibility (range from 1 [completely disagree] to 5 [completely agree])				
8. The TESP intervention seems implementable.	4.25	0.64	3	5
9. The TESP intervention seems possible.	4.40	0.50	4	5

*N* = 21 for questions 1–3; *n* = 20 for questions 4–9

### Rapid Qualitative Analysis

To summarize qualitative interview data, rapid qualitative analysis was applied based on the Rigorous and Accelerated Data Reduction (RADaR) approach (Watkins, 2017). Study co-authors developed an initial thematic framework capturing assessed domains (e.g., A-C/P-C likes and dislikes, implementation barriers and facilitators) and refined it through early transcript review. All interview transcripts and notes were compiled into a spreadsheet serving as the analytic matrix, with rows for participants and columns for specific themes.

Two independent coders identified recurring themes through iterative data-reduction rounds, removing irrelevant content, and retaining key excerpts for thematic coding. Coding discrepancies were reconciled by a third rater. Interrater reliability, calculated using Cohen’s κ, yielded an average of 0.66 agreement (range 0.35–0.90). To reduce redundancy, four overlapping codes were merged into two themes in the presented results. Findings from the qualitative analysis are summarized in Table 2.

**Table 2:** Stakeholder endorsement of themes and supporting quotations, pertaining to adolescent-centered and parent-centered texts as well as implementation barriers and facilitators

Categories	Identified Themes	Supporting Quotations	Stakeholder Endorsement	
			*n*	*%*
Perception of Components				
*Adolescent-Centered Texts Likes & Dislikes*	(+) Content of A-C texts could provide support and be helpful for parents	*“I think that maybe the parents won’t feel so alone with those topics… They’re simple but encouraging*,* and they help them look for things that maybe they’re not thinking about.”*	13	62%
	(+) The mechanics and setup of the A-C texts could be accessible and efficient	*“I like the variety. I like that it’s daily.” “Follow-up calls are helpful*,* but sending regular texts that support the parent and aid in their understanding of the adolescent is hugely valuable.”*	2	10%
	(-) The mechanics and setup of the A-C texts may not be engaging for all parents	*“So in regards to the messages*,* I think it’s good that it’s one-way communication. […] My fear is there’s going to be a desire for two-way communication.” “I might be concerned about the frequency. I can see how that can be a little overwhelming for patients and families.”*	6	29%
	(-) Content of A-C texts may not be helpful for all parents	*“I think a lot of the patients and families we see in the emergency department are quite overburdened and aren’t often looking for alternate means of engagement or support. They are often looking for very specific things*,* whether a specific type of therapy*,* medication*,* or diagnosis.”*	3	14%
*Parent-Centered Texts Likes & Dislikes*	(+) P-C texts are well-aligned with the needs of parents caring for suicidal teens	*“Sometimes parents are so depleted*,* and that reminder that to take care of their child*,* they need to take care of themselves is important.”*	19	90%
	(-) P-C texts may not be well-aligned with the needs of all parents caring for suicidal teens	*“I think the adolescent ones might be a bit more concrete in helping families.”*	5	24%
Implementation Barriers	Sustaining the intervention long-term may be challenging, especially when the emergency department is busy	*“I think our team is already running quite short in terms of our core job responsibilities*,* and that has been a barrier in the past with things we’ve tried to implement clinically.”*	11	52%
	Parents may not be ready or receptive to the texting program	*“I think the first barrier may be a parent focus*,* like do they have the bandwidth to receive the number of text messages and process that information? Is there a point of feeling overwhelmed with that amount of information?” “That could be challenging for a family in the midst of a crisis*,* and I could see that being a barrier.”*	11	52%
	Technology barriers may limit ability for some parents to participate	*“A lot of our patients are in poverty or don’t have access to phones.” “Not everyone uses a cell phone*,* or they might have limitations on data and not want to receive daily text messages because that would raise their bill.”*	9	43%
Implementation Facilitators	Clearly defined staff roles, training, and coordination would be essential for successful program implementation	*“It would need one team dedicated to enrolling patients at some point during their ER process.”*	13	62%
	Health system stakeholders would value clear explanations of the program’s purpose and benefits to facilitate buy-in	*“Helping the group understand how this might improve patient experience*,* safety*,* and some of the values we hold in the ER and health system is important. […] Helping them see the potential impacts and understanding what the intervention is and what it’s doing is key.”*	11	52%
	Parents would value clear explanations of the program’s purpose and benefits to increase interest	*“Having materials*,* visuals that outline what you’re proposing would be really helpful in helping families understand what will happen.”*	9	43%
	Automated and efficient delivery systems would reduce staff burden and support implementation	*“Signing up a patient for this program should be easy*,* like a click in the medical record program.” “You could [automate intervention onboarding] once the patient’s phone number is registered […] without a person needing to be there.”*	5	24%
	Many parents would welcome ongoing support and resources	*“I don’t know if you have [encountered many] parents where they felt they could use extra support.” “Overall*,* parents have been receptive to resources*,* as we’ve consented families for phone calls*,* and feedback has been positive. The vast majority of patients have agreed to calls*,* so I think people would be on board with it.”*	3	14%

Percentages represent the proportion of stakeholders endorsing each theme. Supporting quotations are illustrative examples of stakeholder perspectives

## Results

### Quantitative Feedback

The self-report surveys revealed that, on average, participants held favorable views toward the conceptualization of both the adolescent‑ and parent‑centered components. Specifically, average likability of A-C texts and P-C texts was M = 4.62 (SD = 0.50) and M = 4.48 (SD = 0.87), respectively, on a 5-point scale (1 = dislike a lot, 5 = like a lot). Further, self-reported ratings of intervention feasibility and acceptability (5-point scale, from 1 = completely disagree to 5 = completely agree) were moderately high across indicators, including appeal (M = 4.65, SD = 0.49), perceptions of implementability (M = 4.25, SD = 0.64), and applicability (M = 4.45, SD = 0.60). See Table 1 for full descriptive statistics.

### Qualitative Feedback

#### Intervention Strengths

Participant input concerning the two intervention components suggested an overall positive impression of content relevance and fit. First, the focus of A-C content, which offers practical tips for supporting at-risk adolescents, was viewed by most as beneficial (*n* = 13, 62%). Participants appreciated its focus on concrete safety recommendations (e.g., warning signs, communication, lethal-means restriction). A few (*n* = 2, 10%) explicitly highlighted the variety and efficiency of automated messages focusing on youth safety as a scalable caregiver support tool. Second, the P-C component was widely perceived as well-aligned with parents’ needs to attend to their own distress and needs (*n* = 19, 90%), emphasizing P-C texts may address a key gap by focusing on caregivers’ well-being directly.

#### Intervention Limitations

Participants also recognized some limitations of the intervention components. Concerns related to A-C content delivery mechanics were noted by some (*n* = 6, 29%). For example, 3 participants highlighted potential limits of the intervention’s one‑way communication, expressing that parents may desire two‑way communication, while others indicated that daily frequency of texts may overwhelm families. A small subset of interviewed participants noted that the content of A-C messages might not be helpful for all parents (*n* = 3, 14%), such that caregivers may be more interested in concrete treatment and diagnostic guidance rather than suicide-specific A-C recommendations that are nevertheless more general. Similarly, with respect to P-C messages, some of the participants observed that content focusing on parents’ well-being may feel less relevant for some caregivers (*n* = 5, 24%), noting that some parents may prefer to prioritize their youth’s safety and treatment needs.

#### Implementation Barriers

Key implementation barriers noted by the participants were grouped into three themes (see details in Table 2). First, sustaining implementation in context of competing clinical demands was the most frequently endorsed barrier (*n* = 11, 52%), reflective of limited time and resources in busy EDs. Second, interviewed participants noted two different parent-specific barriers to implementation: while some caregivers may not embrace additional support or lack trust in the system or technology (*n* = 11, 52%), others may experience technology accessibility challenges, such as limited phone access and/or technology literacy (*n* = 9, 43%).

#### Implementation Facilitators

Participants endorsed facilitators centered primarily on system-level organizational structure, workflow automation, and increasing perception of value and buy-in from both staff and parents. First, the most frequently cited facilitator (*n* = 13, 62%) was the need for role clarity and coordination, particularly assigning specific staff (e.g., social workers) to manage intervention roll-out and delivery. Second, participants emphasized the importance of promoting engagement and buy-in from both health system staff (*n* = 11, 52%) and parents (*n* = 9, 43%) alike, such as by clearly communicating the intervention’s purpose and benefits. For example, staff training and linking the intervention’s purpose to supporting patient care and promoting suicide-prevention efforts were seen as key for system-level engagement. Next, participants noted that the automated delivery of the intervention along with automating sign-up features and integration into the electronic medical record (*n* = 5, 24%) would facilitate implementation and scalability.

## Discussion

This study’s purpose was to gather key health system stakeholders’ input on a novel text-based intervention, TESP, designed to facilitate parental engagement in suicide-prevention strategies after their youth’s ED visit. Overall, the interviewed ED providers and administrators/leaders viewed TESP positively as a scalable post-discharge support tool for caregivers of suicidal adolescents. Many emphasized its relevance for parents of at-risk youth, including adolescent-focused messages that reinforce safety recommendations and parent-centered content that validates caregiver experiences. The open-ended sentiments were echoed in positive quantitative ratings of TESP’s components and its potential applicability if integrated into routine ED care. These findings align with broader efforts to develop scalable interventions addressing growing mental health needs of youth seen in healthcare settings (McGorry et al., 2022) as well as digital parenting interventions that help caregivers recognize their own needs and clarify their supportive role (Cao et al., 2025).

With respect to the study’s goal to assess TESP’s implementation potential, quantitative ratings suggest this text-based intervention was seen as implementable if part of routine care. Participating ED providers and administrators/leaders also provided valuable input on specific implementation barriers and facilitators to inform its future adoption. A key system-level barrier centered on sustaining the intervention amid high clinical demands, reflecting real-world challenges of delivering mental health services in busy, resource-strained EDs. Providers and administrators/leaders also identified two implementation barriers specific to parents: lack of trust in the system or technology and limited technology literacy and access. While digital interventions may ease some implementation challenges (e.g., delivery ease, reduced staff burden), these themes suggest that technology can introduce its own barriers (e.g., consumer trust). Of note, participants offered input on implementation facilitators that may help address these and related barriers, including promoting engagement and buy-in from caregivers and health system staff by discussing the intervention’s purpose and potential benefits. Understanding the value of text-based support may encourage caregiver participation, while health system staff buy-in could be strengthened through training and alignment with larger health system-level commitment to improving patient care and suicide-prevention efforts.

The most frequently endorsed implementation facilitator emphasized by participants, however, was that successful implementation requires clear role assignment to ensure longer-term sustainability, which is critical in fast-paced EDs where acute clinical duties take priority. While automation of the text-based intervention and workflow was described as another facilitator, the human aspect related to implementation success for digital interventions cannot be overlooked. Prior work in digital mental health supports this: for example, integration into clinical workflows, referral pathways, role clarity, and staff resources are repeatedly called out as key implementation strategies (Bailey et al., 2024; Graham et al., 2020).

Of importance, health system stakeholders’ feedback reported in this study aligns with prior work showing that brief, automated text-based interventions tend to be seen as feasible and acceptable following a suicidal crisis. In the TESP intervention pilot, post-intervention feedback from parents suggested satisfaction and high acceptability, with the vast majority of parents indicating they would recommend it to parents of high-risk youth (Czyz et al., 2025). Similarly, others have found that hospital-based texting programs demonstrated engagement and satisfaction among adolescents and caregivers (Cancilliere et al., 2024; Suffoletto et al., 2021; Thomas et al., 2024). Here, findings suggest that key health system stakeholders who care for adolescents at risk for suicide and their families similarly view text-based approaches like TESP as an acceptable and feasible way to extend ED care to improve post-discharge safety of youth at elevated suicide risk. More broadly, these findings underscore the importance of incorporating perspectives of health system providers and decision makers when considering opportunities as well as challenges that may arise when introducing digital interventions into complex clinical environments, such as the ED. For example, such feedback may be useful in highlighting how intervention design decisions (e.g., structure, features, delivery mechanics) may influence buy-in and workflow demands in a given setting that can enhance or slow its adoption into routine care. This is in line with the broader implementation science literature that emphasizes incorporating end-user feedback during the pre-implementation phase as a key strategy to promote uptake and sustainability (Dopp et al., 2019; Lyon et al., 2020).

This study’s findings should be interpreted considering several limitations. Data were collected from a small sample of ED staff in two Midwestern health systems, limiting generalizability. Future research should assess real-world uptake across diverse EDs as well as obtain input from a broader range of implementation decision makers and leaders. Although the current study focused on implementation barriers and facilitators of a parent-facing TESP intervention in an ED setting, which shaped the interview guide and discussions, the current study nevertheless may offer key health system leadership and staff insights on acceptability and potential for integrating digital interventions focused on suicide prevention into routine care. More broadly, TESP serves as an example of an automated, ‘set-it-and-forget-it’ digital interventions needed to seamlessly deliver evidence-based suicide prevention recommendations in fast-paced, resource-limited ED settings without placing additional burden on front-line providers.

## Data Availability

Not Applicable.

## Declarations

### Clinical trial number

NCT06660199.

### Author areas of interest

Adaptive digital intervention optimization, equitable care implementation, stakeholder engagement.
